# Light Affects the Chloroplast Ultrastructure and Post-Storage Photosynthetic Performance of Watermelon (*Citrullus lanatus*) Plug Seedlings

**DOI:** 10.1371/journal.pone.0111165

**Published:** 2014-10-23

**Authors:** Qingqing Duan, Wu Jiang, Ming Ding, Ye Lin, Danfeng Huang

**Affiliations:** 1 Department of Plant Science, School of Agriculture & Biology, Shanghai Jiao Tong University, Shanghai, China; 2 Key Laboratory of Urban Agriculture (South), Ministry of Agriculture, Shanghai, China; 3 Department of Horticulture, Northwest A&F University, Yangling, China; Louisiana State University and A & M College, United States of America

## Abstract

Watermelon [*Citrullus lanatus* (Thunb.) Matsum. and Nakai] plug seedlings were stored at 15°C in the light at a photosynthetic photon flux density of 15 µmol·m^−2^·s^−1^ or in darkness for 6 days, to evaluate their chloroplast ultrastructure, and associated photosynthetic characteristics. Storage in the dark caused swelling, disordered granal arrangement, and starch grain disappearance in the chloroplasts. In contrast, the chloroplasts stored in the light were relatively normal. As a result, the light-stored seedlings had a significantly higher chlorophyll content, Fv/Fm, and Pn than did dark-stored seedlings. Regardless of whether the seedlings were stored in light or darkness, the Gs and Ls of the seedlings significantly decreased, while the Ci obviously increased when the Pn decreased after 6 days of storage. This result suggests that the decreased Pn is not solely a stomatal effect, as the effects on the chloroplasts contributed to this photosynthetic inhibition. Six days after transplanting, seedlings that were stored in the light or darkness for 2 or 4 days showed complete recovery of chloroplast ultrastructure, chlorophyll content, Fv/Fm, Gs and Pn. When the storage period increased to 6 days, the dark-stored seedlings had a significantly lower Fv/Fm and Pn than the light-stored and control seedlings 6 days after transplanting, which was mainly ascribed to incomplete recovery of chloroplast ultrastructure. Furthermore, the light-stored seedlings exhibited a significantly higher shoot dry weight during storage and a higher percentage dry weight increase after transplanting than the dark-stored seedlings. These effects were enhanced by prolonged storage (4 to 6 days). This study demonstrated that dim light during storage is beneficial for maintaining chloroplast ultrastructure as well as photosynthetic efficiency in watermelon seedlings, thus contributing to the rapid recovery of post-storage photosynthetic performance, which ensures the transplant quality of the seedlings after removal from storage.

## Introduction

Watermelon [(*Citrullus lanatus* (Thunb.) Matsum. and Nakai)] is a major cucurbit crop worldwide. China is the largest watermelon producer, providing almost two-thirds of the world supply [1]. In China, watermelon is mainly produced in industrial seedling nurseries. To ensure an adequate supply of seedlings, the plants are occasionally stored (e.g., when waiting for optimal weather for transplantation, during shipping, or during labor shortages). Seedlings are normally stored at low temperature in darkness [2]–[4], which can decrease seedling quality. When the plants are stored in darkness, photosynthesis ceases, but respiration continues, depleting the carbohydrate reserves and triggering a series of changes in cellular events, resulting in dry weight loss, decreased chlorophyll content [5], [6], reduced soluble sugars [7], [8], and enhanced lipid peroxidation [9], [10].

The provision of light, even at a very low intensity, during low temperature storage can improve the quality of many horticultural species [8], [11]–[13]. Kubota and Kozai found that the photosynthetic ability of broccoli plantlets that were stored in the light was preserved [5]. Similar results were also observed in seedlings of *Phalaenopsis* [14] and radiata pine [15]. The recovery of post-storage photosynthetic performance is crucial for plants [4], [16], and the post-storage photosynthetic rate is positively associated with new root growth of radiata pine seedlings [15] and survival of Douglas fir seedlings in a greenhouse [17].

However, minimal attention has been focused on how light affects the photosynthetic apparatus of plants during storage and the subsequent recovery period. Plants photosynthetically acclimate to various light conditions through both anatomical and physiological changes [18]–[20]. Chloroplasts are the sites of photosynthesis, and the chloroplast ultrastructure provides a structural framework for photosynthetic performance. Decreases in photosynthesis mainly correspond with ultrastructural alterations in the chloroplast [21]–[23].

We performed experiments to quantify how short-term storage in light or darkness at 15°C and durations from 0 to 6 d influence the chloroplast ultrastructure and photosynthetic status during storage and the subsequent recovery of watermelon seedlings. The specific objectives of this study were (1) to identify the effect of the storage duration and condition (light or darkness) on the chloroplast ultrastructure, chlorophyll fluorescence parameters and gas exchange measurements during storage and (2) to evaluate how storage influences the photosynthetic performance and dry weight accumulation of seedlings after transplanting. Accurately assessing these effects is essential for maintaining watermelon seedling quality during storage and for improving the capacity of photosynthetic recovery after removal from storage.

## Materials and Methods

### Plant Material and Growth Conditions

Seeds of watermelon [*Citrullus lanatus* (Thunb.) Matsum. & Nakai] cv. Zaojia 84–24 were sown in 72-cell plastic plug trays (50 cm^3^ per cell) that were filled with a substrate consisting of peat, perlite, and vermiculite (9∶3:1, v/v/v) in a greenhouse under a natural photoperiod with day/night temperatures of 24/17°C ±3°C. At 20 d after sowing, uniform seedlings with one fully expanded true leaf and one small true leaf were selected and transferred to growth chambers (Model HP GS1000-D, Wuhan Ruihua Instrument & Equipment Co., Ltd., Wuhan, China). The conditions in the growth chambers were as follows: 300 µmol·m^−2^·s^−1^ photosynthetic photon flux density (PPFD) under a 12-h photoperiod, 25/18°C ±2°C day/night, and 70±10% relative humidity. The plants were supplied daily with half-strength Hoagland’s solution.

### Storage and Transplant Conditions

The storage period began when two true leaves had fully expanded and when one small true leaf was in the process of expanding. Six chambers, including twelve plug trays of seedlings, were used in this experiment. There were six trays in three chambers per treatment, providing 432 seedlings per treatment. Two trays in one chamber with 144 seedlings were considered as replications for each treatment. The air temperature inside the chambers was set at 15±1°C, which was recommended as the optimal storage temperature for watermelon plug seedlings [24]. The seedlings were stored for 6 d in darkness or under light at an average PPFD of 15±0.5 µmol·m^−2^·s^−1^. Light was provided during storage by cool white light lamps (Philips LIFEMAX, TLD 30w/865, Royal Philips Electronics, Amsterdam, the Netherlands) that were positioned approximately 30 cm above the seedling canopy. The light compensation point is the optimum PPFD for the low-temperature storage of seedlings [12]. Because there is no reported recommended light intensity for cucurbit storage, the light compensation point for watermelon was estimated as ∼15 µmol·m^−2^·s^−1^ PPFD according to the results of Yongjian [25]. Three additional trays were left under normal growth conditions without storage and served as the non-stored controls. Every 2 d, the seedling trays were taken from the chambers and subirrigated with the same nutrient solution as that used before storage for 5 min under dim light conditions (< 1 µmol·m^−2^·s^−1^) at ambient room temperatures. Then, the trays were relocated to different chambers to minimize environmental differences.

Upon removal from storage at 2, 4, or 6 d, the seedlings from two trays in each treatment were transplanted into 20-cm-diameter plastic pots and allowed to recover in growth chambers under normal culture conditions (25/18°C ±2°C (day/night), PPFD = 300 µmol·m^−2^·s^−1^) for 6 d. The seedlings were supplied with half-strength Hoagland’s solution once per day.

The seedlings were sampled just before the start of storage (0 d) and 2, 4, and 6 d later. After storage in light or darkness, the seedlings were allowed to recover for 6 d and sampled again. In this experiment, measurements were obtained using the first true leaves that were considered mature.

### Electron Microscopy Studies

To avoid variation due to the differential structure in different parts of the leaves, the middle part of the leaves without the midrib was used and cut into small pieces (approximately 12 mm). These small pieces were placed in a bottle with 2.5% glutaraldehyde in phosphate buffer (pH 7.2), and the air was pumped out of the bottle with a syringe to ensure that the leaves would become fully soaked in the buffer solution according to the method of Chen [26] with some modifications. The leaves were fixed at 4°C for 24 h and post-fixed in 2% OsO_4_ (w/v) for 2 h with the same buffer at 4°C. The fixed samples were washed in buffer (three times for 15 min each) and dehydrated in an ascending alcohol series at 4°C: 50% for 15 min, 70% (containing 2% uranyl acetate) for 12 h, 70% for 15 min, and 90% for 15 min. The samples were subsequently washed in steps of 20 min in 90% alcohol/90% acetone (1∶1), 90% acetone at 4°C and in 100% acetone (three times) at room temperature. Then, the samples were immersed in acetone-Epon 812 resin at a ratio of 1∶1 for 4 h and subsequently at a ratio of 1∶2 overnight followed by immersion in 100% resin (three times for 4 h each) and polymerization at 60°C for 48 h. Thin sections (approximately 70 nm thickness) were obtained using a Leica EM UC6 ultramicrotome (Leica Co., Austria) and double-stained with uranium acetate-lead citrate before being examined with a Tecnai G2 Spirit BioTWIN (FEI Company, Hillsboro, Oregon, USA) transmission electron microscope operating at 120 kV.

### Pigment Analysis and Chlorophyll Fluorescence Measurements

The middle part of the first true leaves without the midrib was used to measurechlorophyll content. The samples were collected by punching small disks (0.75 cm diameter) from five plants, and ten disks for each treatment were used for the measurements. Chlorophyll was extracted by incubation in 10 mL of 80% acetone for 48 h in darkness. The chlorophyll content was determined by measuring the absorbance at 663 and 646 nm using a UV–vis spectrophotometer (Model: U-2900, Hitachi Co., Tokyo, Japan) and calculated according to Lichtenthaler [27].

The chlorophyll fluorescence parameters were monitored using a portable modulated chlorophyll fluorometer (FMS-2, Hansatech, Norfolk, UK). The initial fluorescence (Fo) and maximum fluorescence (Fm) were measured after dark adaptation for 30 min. The maximum photochemical efficiency of PSII (Fv/Fm) was calculated as Fv/Fm = (Fm-Fo)/Fm. Ten plants from each treatment were measured.

### Photosynthetic Gas Exchange

The net photosynthesis rate (Pn), stomatal conductance (Gs), and intercellular CO_2_ concentration (Ci) were measured using a Ciras-2 portable photosynthesis system (PP Systems, Amesbury, MA, USA) with an LED light source at 800 µmol·m^−2^·s^−1^ (PPFD). The stomatal limitation (Ls) was calculated according the following equation: Ls = 1 - Ci/Ca, where Ci is the intercellular CO_2_ concentration and Ca is the ambient CO_2_ concentration. The leaf area clipped by the chamber was 2.5 cm^2^. The temperature, relative humidity, and CO_2_ concentration were maintained at 25°C, 45±5%, and 375 µmol·mol^−1^, respectively, during the measurements. The measurements were performed on leaves from at least three plants for each treatment during storage and after transplanting.

### Plant Growth Parameters and Tissue Biomass After Transplanting

Twelve plants from each treatment were sampled. The sampled plants were washed carefully to remove the medium, and the shoots were cut from the plants. The washing procedure was performed at room temperature within a few minutes for each plant. Subsequently, the shoots were dried at 80 °C for 48 h in folded paper envelopes to obtain the shoot dry weight. The percentage increase in shoot dry weight for each treatment was measured on the 1^st^ day after removal from storage and the 6^th^ day after transplanting.

### Statistical Analysis

The statistical analyses were performed using SPSS 16.0 for Windows (SPSS, Chicago, IL, USA). The data were subjected to analysis of variance (ANOVA), and the mean values were compared by Tukey’s test (P<0.05) when a significant difference was detected.

## Results

### Changes in Chloroplast Ultrastructure, Chlorophyll Content, Chlorophyll Fluorescence Measurements, and Photosynthesis During Storage in Light or Darkness

#### Changes in The Appearance of Leaves and Ultrastructure of Chloroplasts

**Figure 1:**
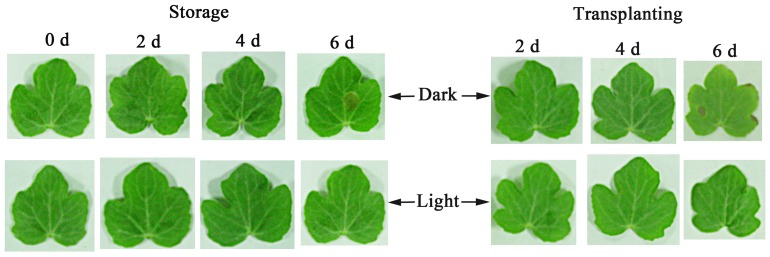
Photographs of the first true leaves of watermelon (*Citrullus lanatus*) seedlings during storage in light or darkness at 15°C and subsequent transplanting. (The seedlings were stored for 2, 4, or 6 d. After removal from storage, the seedlings were transplanted for 6 days.).

**Figure 2:**
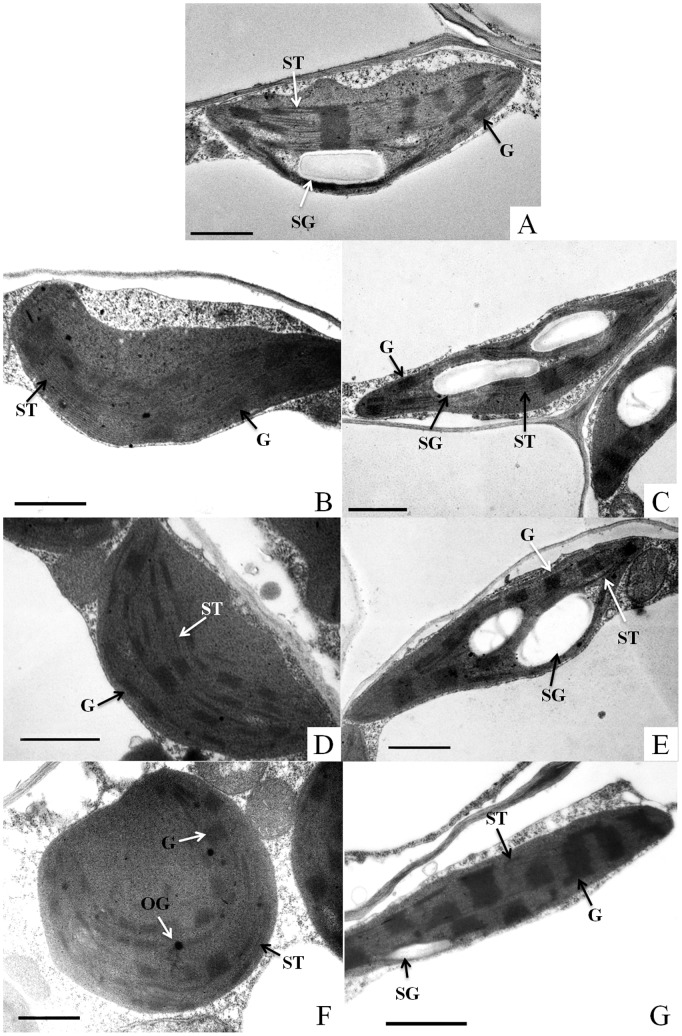
Changes in the ultrastructure of chloroplasts from the leaves of watermelon (*Citrullus lanatus*) seedlings stored in light (C, E, G) or darkness (B, D, F) at 15°C for 0 (A), 2 (B, C), 4 (D, E), or 6 (F, G) days. Bar = 1 µm. Abbreviations: ST, stroma thylakoid; G, grana thylakoid; SG, starch grain; OG, osmiophilic globule.

No obvious differences in leaf appearance were observed between the light and dark treatments within 4 d of storage. However, after 6 d of storage, dark-stored seedlings exhibited some necrosis on the leaves, whereas light-stored leaves appeared vigorous relative to dark-stored leaves (Fig. 1). In normal leaves (0 d), the chloroplasts were ellipsoid in shape, and the grana and stroma lamellae of the chloroplasts were well developed; the thylakoid was arranged densely along the long axis of the chloroplasts, and starch grains were observed (Fig. 2 A). No substantial differences in the chloroplasts were found between light-stored and non-stored seedlings until the 4^th^ day of storage (Fig. 2 C, E). On the 6^th^ day of light storage, the starch grains of the chloroplasts decreased in number and size, and the chloroplasts appeared elongated in shape, with an increased thickness and an increased number of granal thylakoids (Fig. 2 G). In contrast, chloroplasts from the dark-stored leaves showed remarkable differences, with the chloroplast appearing to be swollen during storage (Fig. 2 B, D) and exhibiting a round shape on the 6^th^ day of storage. In addition, some osmiophilic globules were observed in the chloroplasts, and the granal arrangement was distorted, with an obscure boundary between the grana thylakoids and stroma thylakoids (Fig. 2 F). Furthermore, dark storage caused the disappearance of starch grains.

#### Chlorophyll Content and Chlorophyll Fluorescence Measurements

**Figure 3:**
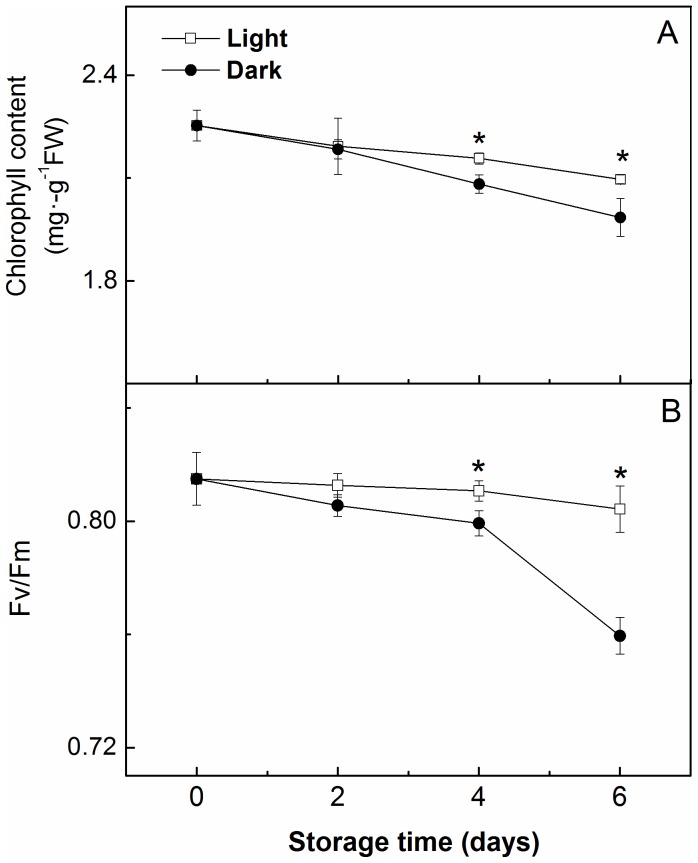
Changes in the chlorophyll content (A) and maximal photochemical efficiency of PSII (Fv/Fm) (B) in the leaves of watermelon (*Citrullus lanatus*) seedlings stored in the light (□) or in the dark (•) at 15°C. Data in A are the means of five replicates, and data in B are the means of ten replicates. Standard errors are shown with a vertical bar. Asterisks indicate significant differences between treatments on a given day according to the Tukey test (P<0.05).

As shown in Fig. 3 A, the chlorophyll content of the leaves decreased with the duration of the storage period under dark conditions and significantly decreased after 6 d of storage in the dark, while the chlorophyll content was maintained at a relatively higher level in the light compared with the darkness. The Fv/Fm is the maximum photochemical efficiency of PSII and represents the function of PSII. Similar to chlorophyll, the Fv/Fm of the seedlings remained relatively unchanged during storage under light conditions but decreased under dark conditions (Fig. 3 B). Moreover, the seedlings that were stored in the light had a higher Fv/Fm as well as a higher chlorophyll content during storage compared with those that were stored in darkness.

#### Photosynthesis

During storage, the Pn, Gs, and Ci of the light-stored and dark-stored seedlings decreased gradually over time; however, the Ls increased until the 4^th^ day of storage (data not shown). In contrast, after 6 d of storage, the seedlings showed a marked decrease in the Pn accompanied by a significant decrease in the Gs and Ls and a remarkable increase in Ci in the leaves compared with the seedlings before storage (Table 1). The seedlings that were stored in the light showed a higher Pn and Gs during storage than those that were stored in darkness.

**Table 1:** The net photosynthesis rate (Pn), stomatal conductance (Gs), intercellular CO_2_ concentration (Ci), stomatal limitation (Ls) of mature leaves, and shoot dry weight of watermelon (*Citrullus lanatus*) seedlings before and after 6 days of storage.

Treatments	Pn (µmol⋅m^−2^⋅s^−1^)	Gs (mmol⋅m^−2^⋅s^−1^)	Ci (µmol⋅mol^−1^)	Ls	Shoot dry weight (g⋅plant^−1^)
Before storage					
	20.0 a	120.1 a	254.7 c	0.56 a	0.114 a
After 6 days of storage					
Light storage	7.5 b	62.8 b	266.9 b	0.47 b	0.105 a
Dark storage	2.7 c	49.7 c	439.8 a	0.14 c	0.087 b

Means in columns followed by different letters are significantly different by Tukey test at P<0.05.

#### Shoot Dry Weight

The shoot dry weight per seedling (the total dry weight of the leaf and stem) after storage in the light for 6 d was not significantly different from that before storage (Table 1), while the shoot dry weight of the seedlings that were stored in darkness for 6 d decreased significantly compared to that of the seedlings before storage and was obviously lower than that of the light-stored seedlings.

### Recovery of Chloroplast Ultrastructure, Chlorophyll Fluorescence, and Photosynthesis After Transplanting

#### Chloroplast Ultrastructure and Leaf Appearance

**Figure 4:**
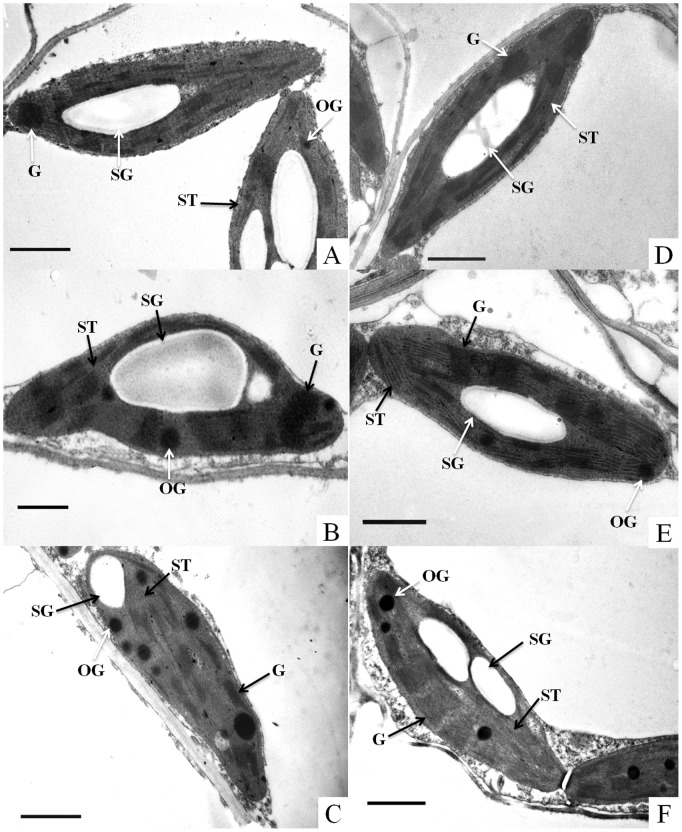
Effect of storage duration on the post-storage ultrastructure of chloroplasts from the leaves of watermelon (*Citrullus lanatus*) seedlings. Seedlings were transplanted for 6 days after being stored in the dark (A, B, C) or in the light (D, E, F) for 2 (A, D), 4 (B, E), or 6 (C, F) days at 15°C. Bar = 1 µm. Abbreviations: ST, stroma thylakoid; G, grana thylakoid; SG, starch grain; OG, osmiophilic globule.

Six days after transplanting following dark storage for 2 or 4 d, the chloroplasts in the leaves returned to a normal oval shape, the thylakoids were orderly and dense, and the starch grains reaccumulated (Fig. 4 A, B). Moreover, there were no differences in the leaf appearance (Fig. 1) or chloroplast ultrastructure compared to light-stored seedlings (Fig. 3 D, E). However, the seedlings that were stored for 6 d in the dark did not recover normal leaf phenotypes or chloroplast ultrastructure by the 6^th^ day of transplanting, showing greater necrosis on the leaves, numerous osmiophilic globules and disordered grana thylakoids in the chloroplasts (Fig. 4 C). In contrast, 6 d after transplantation, the seedlings that were stored in the light for 6 d exhibited healthier leaves (Fig. 1) and had more starch grains and fewer osmiophilic globules in the chloroplasts than dark-stored seedlings (Fig. 4 C). Moreover, no differences were observed in the chloroplast shape (Fig. 4 F) or the thylakoid structure of light-stored chloroplasts compared with normal chloroplasts (Fig. 2 A).

#### Chlorophyll Content and Chlorophyll Fluorescence Measurements

**Figure 5:**
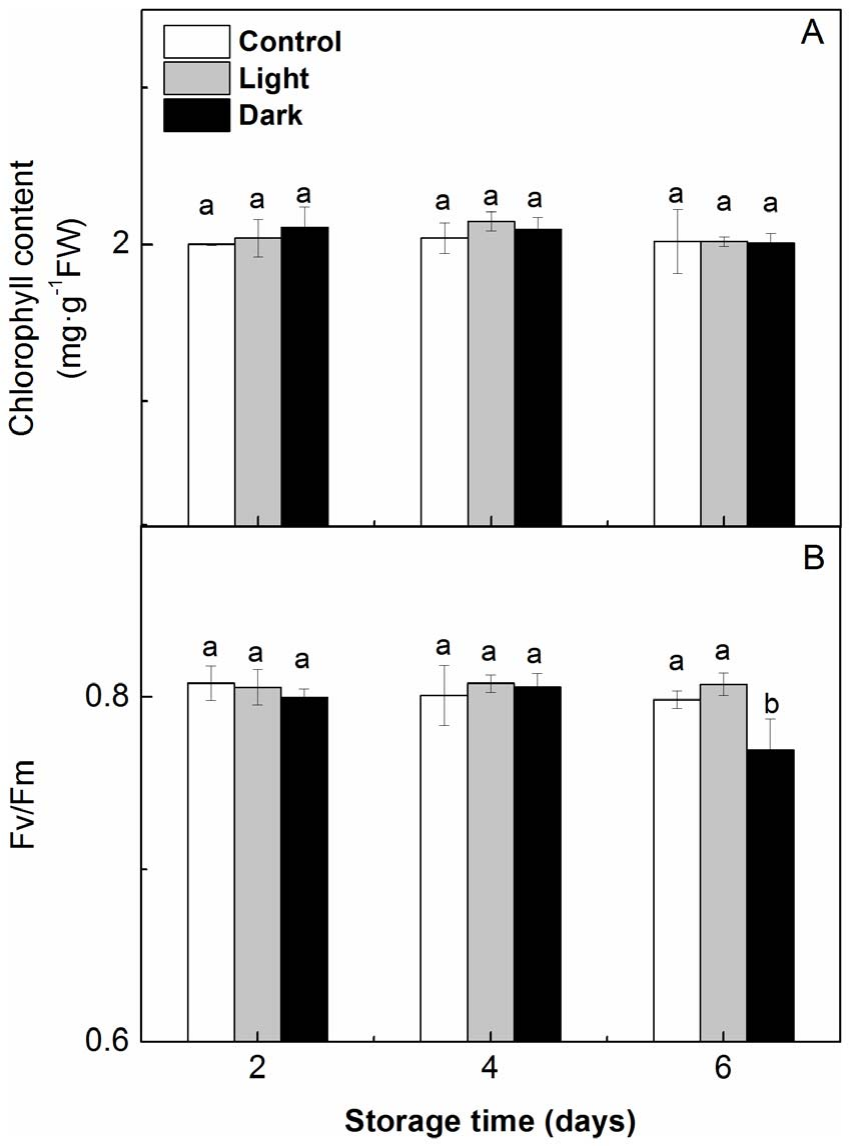
Effect of storage duration on the post-storage chlorophyll content (A) and maximal photochemical efficiency of PSII (Fv/Fm) (B) from the leaves of watermelon (*Citrullus lanatus*) seedlings. Seedlings were transplanted for 6 days after being stored at 15°C in the dark (*black*) or in the light (*gray*) for 2–6 days. The controls (*white*) were taken from seedlings that had never been stored. Data in A are the means of five replicates, and data in B are the means of ten replicates. Standard errors are shown with a vertical bar. Small letters indicate significant differences between treatments on a given day according to the Tukey test (P<0.05).

As shown in Fig. 5, the chlorophyll content and the Fv/Fm of the seedlings that were stored in the light for 2, 4, or 6 d had recovered by 6 d after removal from storage. When the seedlings were stored in the darkness for 2 or 4 d, the chlorophyll content and Fv/Fm also recovered to control (non-stored seedling) levels by 6 d after transplanting. When the duration of dark storage was extended to 6 d, the Fv/Fm of the seedlings was significantly lower than that of the control and light-stored seedlings, but the chlorophyll content recovered to control levels by the 6^th^ day after transplanting.

#### Photosynthesis

**Figure 6:**
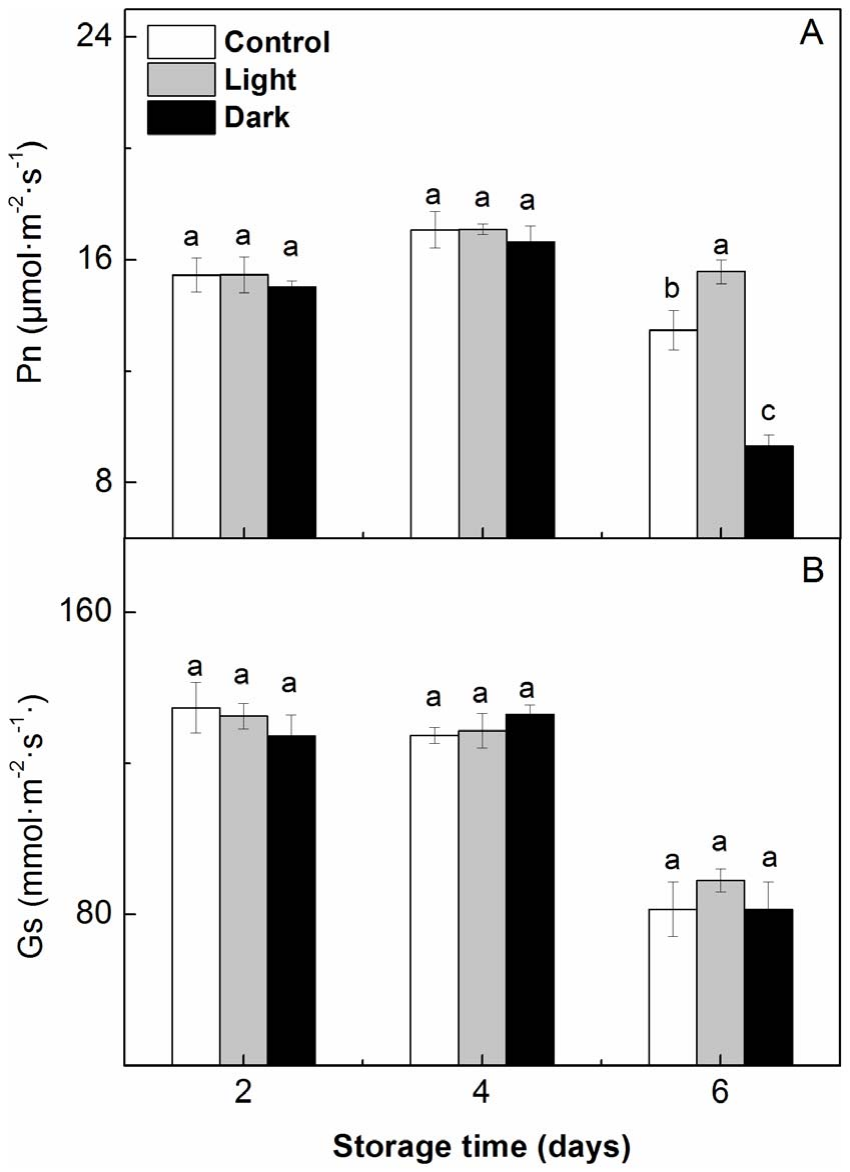
Effect of storage duration on the post-storage net photosynthesis rate (Pn) (A) and stomatal conductance (Gs) (B) in the leaves of watermelon (*Citrullus lanatus*) seedlings. Seedlings were transplanted for 6 days after being stored at 15°C in the dark (*black*) or in the light (*gray*) for 2–6 days. The controls (*white*) were taken from seedlings that have never been stored. Data are the means of nine replicates; standard errors are shown with a vertical bar. Small letters indicate significant differences between treatments on a given day according to the Tukey test (P<0.05).

The post-storage recovery of the Pn varied depending on the duration of storage and the light conditions during storage. As shown in Fig. 6, at 6 d after transplantation, the seedlings that were stored under dark or light conditions for 2 or 4 d showed no differences in Pn or Gs; however, as the storage duration increased to 6 d, the post-storage Pn was significantly higher in the light-stored plants than in the control and dark-stored plants. The Gs of the seedlings at 6 d after transplanting was not influenced by 6 d of storage in light or darkness.

#### Shoot Dry Weight Increase

**Figure 7:**
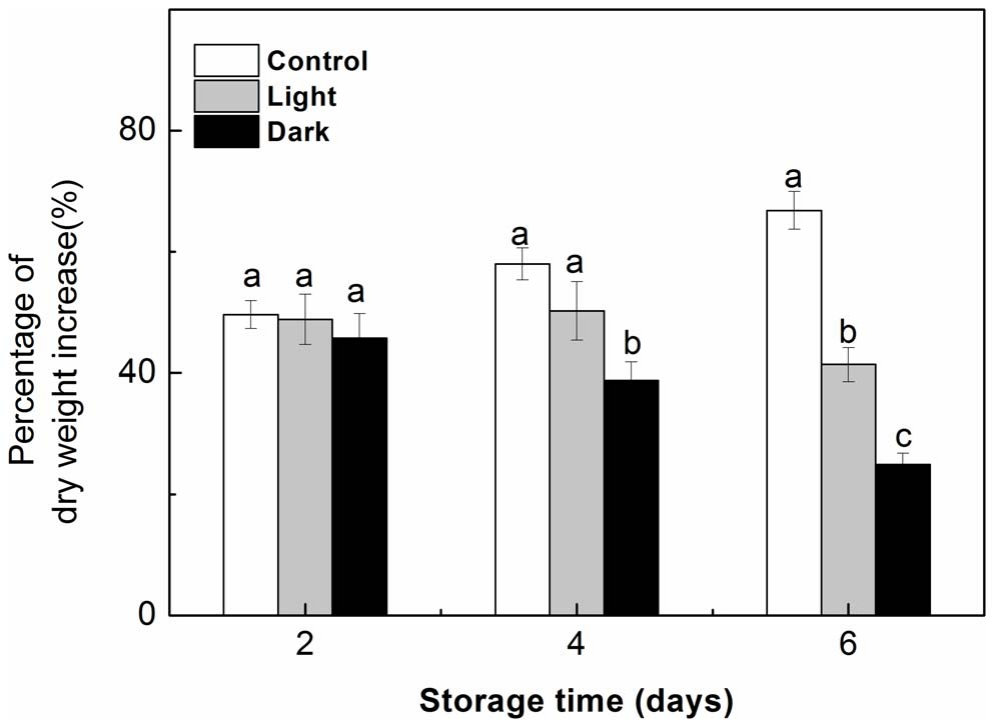
Effect of storage duration on the post-storage percentage dry weight increase in shoots of watermelon (*Citrullus lanatus*) seedlings. Seedlings were transplanted for 6 days after being stored at 15°C in the dark (*black*) or in the light (*gray*) for 2–6 days. The controls (*white*) were taken from seedlings that have never been stored. Data are the means of twelve replicates; standard errors are shown with a vertical bar. Small letters indicate significant differences between treatments on a given day according to the Tukey test (P<0.05).

The seedlings that were stored under light or dark conditions for 2 d exhibited no obvious differences in the percent dry mass increase compared to the control seedlings at 6 d after transplanting (Fig. 7). The percentage dry mass increase of the seedlings that were transplanted after 6 d following storage in the light for 6 d or in darkness for 4 or 6 d was significantly lower relative to that observed in the respective control seedlings. Moreover, the percent dry mass increase in the seedlings that were stored in darkness for 4 or 6 d was significantly lower than that of the light-stored seedlings at 6 d after transplantation.

## Discussion

### Light Preserves the Chloroplast Ultrastructure and Photosynthetic Performance of Watermelon Seedlings During Storage

Chloroplast development depends on light, and light affects chloroplast structure and photosynthetic changes in plants [28], [29]. In this study, dark storage significantly altered the chloroplast ultrastructure of the leaves: the chloroplasts gradually swelled to a rounded shape, and the granal thylakoids were disordered with a low stacking degree (Fig. 2 B, D, F). Similar results were also found in detached barley leaves that were cultured in the dark for 4 d, showing a distorted granal arrangement and an increase in the number and size of plastoglobuli [30]. Furthermore, dark storage caused the disappearance of starch grains, mainly because starch grains serve as a reserve material that can be used to allow plants to survive starvation induced by darkness [31]. In contrast, the chloroplasts from the light-stored leaves were relatively normal, with no differences compared to the chloroplasts observed before storage until the 4^th^ day after storage (Fig. 2 A, C, E). However, on the 6^th^ day, it was noted that the thickness of the granal thylakoids was increased compared with that before storage (Fig. 2 G). This result agrees with earlier reports, which showed that leaves that were grown under low light had thicker grana lamellae and an increased number of granal thylakoids in the chloroplasts than those under normal light intensity; these alterations would improve the light-harvesting ability of the thylakoids, serving as an adaptation to low-light conditions [28], [32], [33]. These results indicate that the starch grains in the chloroplast and the chloroplast ultrastructure of the leaves, including a regular thylakoid arrangement and dense stacking of grana, are maintained during light storage but are destroyed during dark storage.

During dark storage, the chlorophyll in seedlings and leafy vegetables is susceptible to degradation [5], [6]. The degradation of chlorophyll involves alteration of the chloroplast ultrastructure [34]. The considerable alteration of the chloroplast ultrastructure in dark-stored leaves might be the result of their rapid loss of chlorophyll (Fig. 3 A). When the chlorophyll of PSII is destroyed, the Fv/Fm decreases [35]. The present data indicate that the seedlings that were stored in the light had a higher chlorophyll content and higher Fv/Fm (Fig. 3 B) during storage than those that were stored in darkness, indicating that the chlorophyll remaining in the leaves of the seedlings that were stored in the light could facilitate photosynthesis in a relatively efficient manner.

After 6 d of storage, the Pn, Gs and Ls decreased significantly compared to their levels before storage, but the Ci increased remarkably in the leaves of the seedlings that were stored in light or darkness (Table 1); however, the seedlings that were stored in the light had a higher Pn and Gs than those that were stored in darkness. This result indicates that non-stomatal limitations contribute to the storage-induced decrease in Pn, regardless of the light conditions during storage. Chlorophyll breakdown and damage of the chloroplast structure are always accompanied by a decrease in photosynthesis [34]. In this study, the loss of chlorophyll and the alteration of the chloroplast ultrastructure in the leaves might be responsible for the reduction in photosynthesis during storage.

Dry mass is an important factor in plant storage because it indicates how environmental conditions affect the plant growth rate and dry mass accumulation [11]. Illumination during storage helps to maintain the dry weight of plantlets better than dark storage. Compared to that before storage, the shoot dry weight of the seedlings remained relatively unchanged under light storage for 6 d but decreased significantly under dark storage due to the continuous respiration that occurs in darkness (Table 1). Moreover, the seedlings that were stored in the light had a significantly higher shoot dry mass than those that were stored in darkness. The stability of the dry weight during light storage indicates that the PPFD of 15 µmol·m^−2^·s^−1 ^in this study was close to the light compensation point for watermelon seedlings at 15°C.

### Post-storage Photosynthetic Performance is Associated with Recovery of the Chloroplast Ultrastructure and the Duration of Storage

Recovery of the chloroplast ultrastructure in light- or dark-stored seedlings varies among plant species. In conifer trees, including larch, pine, and spruce, 2 d of illumination was adequate for the restoration of chloroplast ultrastructure when seedlings were grown in the dark for 13 d [36], while chloroplasts from the cotyledons of *Cucurbita pepo* that were treated in the dark for 5 d required 5 d of photoactivation to restore their normal shape and thylakoid membrane system [37]. In this study, the seedlings that were stored in the dark for 2 or 4 d recovered a normal chloroplast shape and thylakoid structure 6 d after transplanting, while the seedlings that were stored in the dark for 6 d did not recover their thylakoid structure, with many osmiophilic globules and disordered grana thylakoids (Fig. 4). In contrast, the seedlings that were stored in the light for 6 d completely recovered their chloroplast structure and had fewer osmiophilic globules and more ordered grana in the chloroplasts than the seedlings that were stored in darkness (Fig. 4).

When the storage duration increased to 6 d, the post-storage photosynthesis was less dependent on the recovery of the chlorophyll content and Gs. On the 6^th^ day after transplanting, the chlorophyll content (Fig. 5 A) and Gs (Fig. 6 B) in the seedlings that were stored in light or darkness did not differ from those of the control seedlings, regardless of the storage duration. Although their chlorophyll concentrations and Gs were the same, the light-stored seedlings had a significantly higher Pn than the dark-stored and control seedlings (Fig. 6 A). These data suggest that the pigment concentration and stomatal factors are not the main elements affecting photosynthesis during this stage.

The structure and function of chloroplasts are important for the growth of plants and influence plants’ physiological and ecological responses [38]. It is commonly accepted that a decrease in photosynthesis is related to disturbance of the chloroplast structure [34], [39]. Simultaneously with the change in the chloroplast structure, 6 d after transplanting, the Fv/Fm and Pn of the seedlings that were stored in the light for 2, 4 or 6 d and the seedlings that were stored in darkness for 2 or 4 d were completely restored to control levels (Fig. 5, Fig. 6). However, on the 6^th^ day after removal from storage, the seedlings that were stored in the dark for 6 d had a significantly lower Fv/Fm and Pn than the light-stored and control seedlings, indicating that the Fv/Fm and Pn could not be recovered in the dark-stored seedlings in this time period, mainly due to the incomplete restoration of the chloroplast ultrastructure in these seedlings.

The high photosynthetic ability during storage contributes to the subsequent growth of seedlings after storage [15], [40]. The seedlings that were stored in the light for 6 d appeared vigorous and survived, while the seedlings that were stored in darkness deteriorated due to necrosis or chlorosis on old leaves or cotyledons, resulting in the death of 30% of the seedlings after transplanting (data not shown). The percentage dry weight increase of the seedlings decreased as the storage time increased from 2 to 6 d for the stored seedlings; in contrast, the percentage dry weight increase of the control seedlings continuously increased over time (Fig. 7). These data indicate that the storage duration affects the regrowth potential of seedlings, with a lengthened storage period causing a decrease in the percentage dry mass increase after transplanting, regardless of whether the seedlings were stored under light or dark conditions. However, the light-stored seedlings had a higher regrowth ability than the dark-stored seedlings after removal from storage.

The purpose of seedling storage is to stop or suppress the growth and development of seedlings while preserving their quality and not adversely affecting their future growth. Photosynthetic ability is a good indicator of the visual quality of seedlings during storage [15], [41] and the growth potential of seedlings after transplanting [40]. In this experiment, the watermelon seedlings that were stored in the light had a higher Pn and Fv/Fm than those that were stored in darkness (Table 1 and Fig. 3), which contributed to the preservation of the shoot dry mass of seedlings (Table 1). After transplanting, the light-stored seedlings had a higher percentage dry weight increase (Fig. 7) and exhibited faster recovery of their photosynthetic ability than the dark-stored seedlings; 6 d was adequate for the recovery of the Pn and Fv/Fm, even when the storage time was extended to 6 d (Fig. 5 and Fig. 6). In this study, the quality of the light-stored watermelon seedlings might have been higher because 1) the maintenance of the chloroplast ultrastructure with densely stacked grana in the leaves during light storage contributed to photosynthesis; 2) the higher chlorophyll concentration and the higher Fv/Fm of PSII in the seedlings that were stored in the light reduced damage to PSII reaction centers; 3) the rapid recovery of the chloroplast ultrastructure and photochemical activities had beneficial effects on photosynthesis after removal from storage; or 4) the increased vigor of the seedlings that were stored in the light increased the dry weight during storage and enhanced the regrowth potential after transplanting.

## Conclusions

This study demonstrated the effects of light during storage and the storage duration on the photosynthetic apparatus and its ability in watermelon seedlings. The current data indicate that the seedlings that were stored at 15°C in the light exhibited a normal chloroplast ultrastructure with well-organized grana thylakoids and showed an improvement in photosynthetic performance with a significantly higher Pn and Fv/Fm compared with the dark-stored seedlings. Six days might be adequate for the post-storage recovery of Pn and Fv/Fm, but the effect depends on the light condition and the duration of storage. Thus, the seedlings that were stored in the darkness for 6 d appeared to require a longer time period to recover their photosynthetic ability. Furthermore, the percentage dry mass increase was greatly reduced when the seedlings were stored in the dark, and this effect was worsened by prolonged storage (4 to 6 d). This study indicates that dim light (PPFD = 15 µmol·m^−2^·s^−1^) during storage is beneficial for maintaining the chloroplast ultrastructure and the photosynthetic efficiency in watermelon seedlings, thus contributing to the rapid post-storage recovery of photosynthetic performance, which ensures the transplant quality of the seedlings after removal from storage.
